# The Roles of Pseudophosphatases in Disease

**DOI:** 10.3390/ijms22136924

**Published:** 2021-06-28

**Authors:** Andrew M. Mattei, Jonathan D. Smailys, Emma Marie Wilber Hepworth, Shantá D. Hinton

**Affiliations:** Integrated Science Center, Department of Biology, College of William and Mary, Williamsburg, VA 23185, USA; ammattei@email.wm.edu (A.M.M.); jdsmailys@email.wm.edu (J.D.S.); ewhepworth@email.wm.edu (E.M.W.H.)

**Keywords:** pseudoenzymes, pseudophosphatases, protein tyrosine phosphatases (PTPs), dual specificity phosphatases (DUSPs), myotubularin phosphatases (MTMs), STYX (phosphoserine/threonine/tyrosine-interacting protein), MK-STYX (MAPK (mitogen-activated protein kinase) phosphoserine/threonine/tyrosine-binding protein), tensin, disease

## Abstract

The pseudophosphatases, atypical members of the protein tyrosine phosphatase family, have emerged as bona fide signaling regulators within the past two decades. Their roles as regulators have led to a renaissance of the pseudophosphatase and pseudoenyme fields, catapulting interest from a mere curiosity to intriguing and relevant proteins to investigate. Pseudophosphatases make up approximately fourteen percent of the phosphatase family, and are conserved throughout evolution. Pseudophosphatases, along with pseudokinases, are important players in physiology and pathophysiology. These atypical members of the protein tyrosine phosphatase and protein tyrosine kinase superfamily, respectively, are rendered catalytically inactive through mutations within their catalytic active signature motif and/or other important domains required for catalysis. This new interest in the pursuit of the relevant functions of these proteins has resulted in an elucidation of their roles in signaling cascades and diseases. There is a rapid accumulation of knowledge of diseases linked to their dysregulation, such as neuropathies and various cancers. This review analyzes the involvement of pseudophosphatases in diseases, highlighting the function of various role(s) of pseudophosphatases involvement in pathologies, and thus providing a platform to strongly consider them as key therapeutic drug targets.

## 1. Introduction

Pseudoenzymes’ inability to perform catalysis categorizes them as atypical members within their superfamilies [1,2,3,4,5,6]. Over the past decade, the relevance of pseudoenzymes has been elevated and solidified as important signaling regulators. Studies have revealed their function in signaling cascades and some of their modes of action. Among these, pseudoenzymes, pseudokinases and pseudophosphatases provide the best examples of their molecular mechanisms. They serve as competitors, signal integrators, modulators, and anchors in cellular processes [5,6,7]. These modes of action and their roles in various cellular processes serve as an idea link for the roles of pseudoenzymes in pathologies and diseases. Misregulation of pseudoenzymes has been implicated in the etiology of various diseases such as cancer, obesity, and neurological disorders [4,5,7,8,9]. Thus, these molecules are excellent and promising candidates for therapeutic drug targets. This review highlights the signaling pseudophosphatases and their implications in diseases, while demonstrating some of their essential roles in preventing or causing such diseases.

### 1.1. Pseudophosphatases

Pseudophosphatases of PTPs are widely accepted as having mutations within their signature active site motif (HCX_5_R) that renders them inactive [4,6,10,11,12,13]. Mutations also may exist beyond the active site motif. Pseudophosphatases are not limited to the protein tyrosine phosphatase family, but are among the serine/threonine phosphatases as well [5,14,15]. They maintain the three-dimensional fold and the ability to bind phosphorylated proteins [4,11,16]. Recent reports have advanced the field, complicating the mutated sequence definition [11,17] and the “grab and hold on” perspective, where pseudophosphatases stably interact with phosphorylated residues, thereby competitively inhibiting active phosphatases or kinases [11,16]. They bind such residues; however, some have been reported to perform catalysis [5,17]. For example, the histidine domain containing protein tyrosine phosphatase (HD-PTP) is reported as a pseudophosphatase [4,5,18,19,20,21,22] and an active phosphatase [4,5,14,23,24]. It has a mutation in the codon for aspartic acid 181 [4,5,18,24], the position that is critical for PTP to serve as a general acid in the first step of catalysis [4,24]. Thus, it is classified as a pseudophosphatase [13], and there are studies that demonstrate that HD-PTP functions as a pseudophosphatase in the endosomal sorting pathway [5,21].

This dual and contradictory functionality of HD-PTP highlights the complexities of investigating pseudophosphatases. This complexity also lies in defining pseudoenzymes through a solely bioinformatics approach [5]. Wishart and Dixon defined pseudophosphatases as STYX domains, and demonstrated a loss of function of dephosphorylating phosphorylated substrates [12]; thus, they defined the prototypical STYX domain beyond pure bioinformatics [12]. The analysis of pseudophosphatases through a combination of sequencing and functional experiments—dephosphorylation assays with appropriate substrates and contextual situations (localization, substrates/interactors, or abundance of interactor or pseudphosphatase—will provide more of an insight into the full spectrum [4,5,7]. Nevertheless, the roles of pseudophosphatases as regulators in many cellular processes such as spermatogenesis, stress response, neuronal differentiation, cell fate, migration, ubiquitylation, demyelination, oocyte-to-zygote transition, transcription, and apoptosis [4,6,7,16,20,22,25,26] have led to interest into their apparent roles in diseases.

### 1.2. Pseudophosphatases in Diseases

The increased interest in pseudophosphatases resulted in the discovery of their relevance in many diseases. Reiterer et al. provided a useful framework for pseudophosphatases’ linkage to diseases [7]. Many recent studies have demonstrated an expansion of these linkages and the possible modes of action for various pseudophosphatases. Pseudophosphatases are important regulators, and their proper regulation is important for maintaining homeostasis and preventing diseases [5]. Mutations of these molecules or their misregulation leads to diseases such as leukemia, breast cancer, colorectal cancer, hepatocarcinoma, glioblastoma, other cancers, Charcot-Marie-Tooth (CMT) disease (abnormal nerve myelination), obesity, diabetes, chronic obstructive pulmonary disease (COPD), and nephrotic syndrome [5,7,27,28,29,30,31,32,33]. A comprehensive list of the known functions of pseudophosphatases and their implications in diseases is provided in Table 1. It is apparent that they are implicated in many diseases (Table 1).

Furthermore, those that have not been linked to diseases have functions that may allude to a possible role in diseases. The diversity of these diseases, such as various types of cancer, COPD, CMT, obesity, diabetes, etc. (Table 1), makes it challenging to discuss every single disease linkage with pseudophosphatases. Furthermore, their diverse range, while validating the intricate roles of pseudophosphatases in signaling cascades, also makes understanding their many functions in diseases more complicated. Considering this special edition focused on the roles of protein tyrosine phosphatases in signaling, with an emphasis on therapeutic strategies, the pseudophosphatases’ signaling mechanisms linked to diseases, myotubularins, tensins, and STYX pseudophosphatases, was discussed in more detail.

## 2. Myotubularin in Diseases

**Figure 1:**
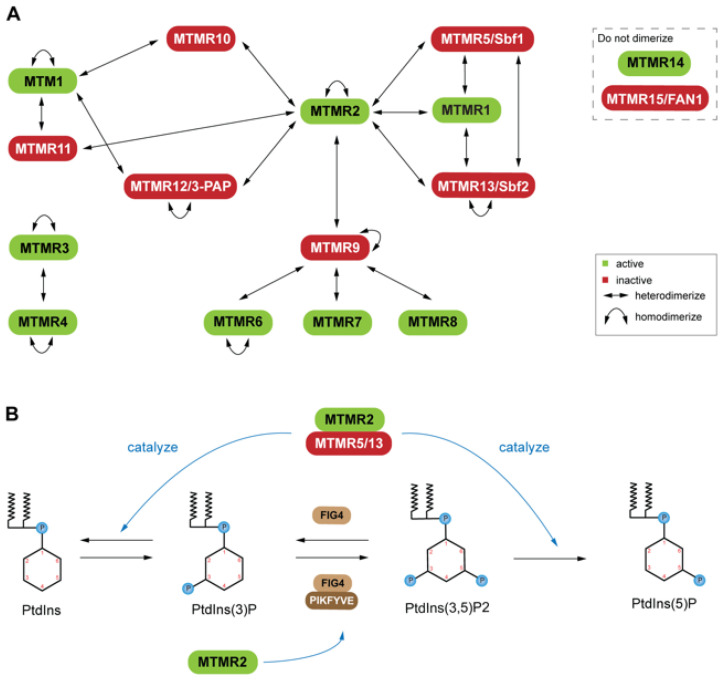
Dimerization states of the myotubularin (MTM) family: (**A**) Active and catalytically inactive forms are indicated in green and red, respectively. Active and inactive coupling (heterodimerization) is common; however, self-association (homodimerization) has also been reported among both active and inactive MTMs. MTMR14 and MTMR15 are not known to dimerize. (**B**) Phosphorylation schema for phosphatidylinositol (PtdIns), a molecule important for endosomal-lysosomal membrane trafficking, with example PtdIns kinases (PIKFYVE) and non-MTM phosphatases (FIG4), along with active and inactive MTMs (labelled green and red, respectively). Heterodimerization of an active (MTMR2) and inactive (MTMR5, MTMR13) leads to a stabilized complex with PtdIns phosphatase potential. MTMR2 and FIG4 interactions have been implicated in vivo, though further characterization is necessary.

Myotubularins (MTMs), the most prevalent of the pseudophosphatases [10,27,69,70], consist of fourteen genes in the human [27,71,72]. Six of these genes produce pseudophosphatases, which form complexes with their active homologs [27,71,72] (Figure 1A). Inactive MTMs serve as scaffolds, complexing with the active enzymes. This dimerization is context dependent, resulting in complex stability, enhancement of catalytic function (increased phosphatase activity), and regulation of the subcellular localization of the active phosphatase [73,74]. The coupling of active and inactive MTMs to form heterodimers is common; however, self-association to form homodimers also occurs among both active and inactive MTMs (Figure 1A) [5,72,75]. MTMR13/Sbf (Set-binding factor 2) and MTMR2 homodimers are thought to associate with one another to form tetramers [75]. Both dimerization events occur through a coiled-coil domain that is conserved within the MTM family. It is of interest that some MTMs have not been reported to interact directly as dimers [72]. These uncoupled MTMs consist of an active PTP (MTM14) and a pseudophosphatase (MTM15/FAN) (Figure 1A) [72]. The heterodimers provide an excellent example of the significance of both the active MTM and the catalytically inactive MTM’s important roles as regulators. MTMs have been linked to several neuropathies [30,71,76]. Mutations of MTM-related protein 2 (MTMR2; (phosphatase)) and MTMR13 (pseudophosphatase) involvement in diseases suggests that this phosphatase-pseudophosphatase complex has a very important role in disease.

**Figure 2:**
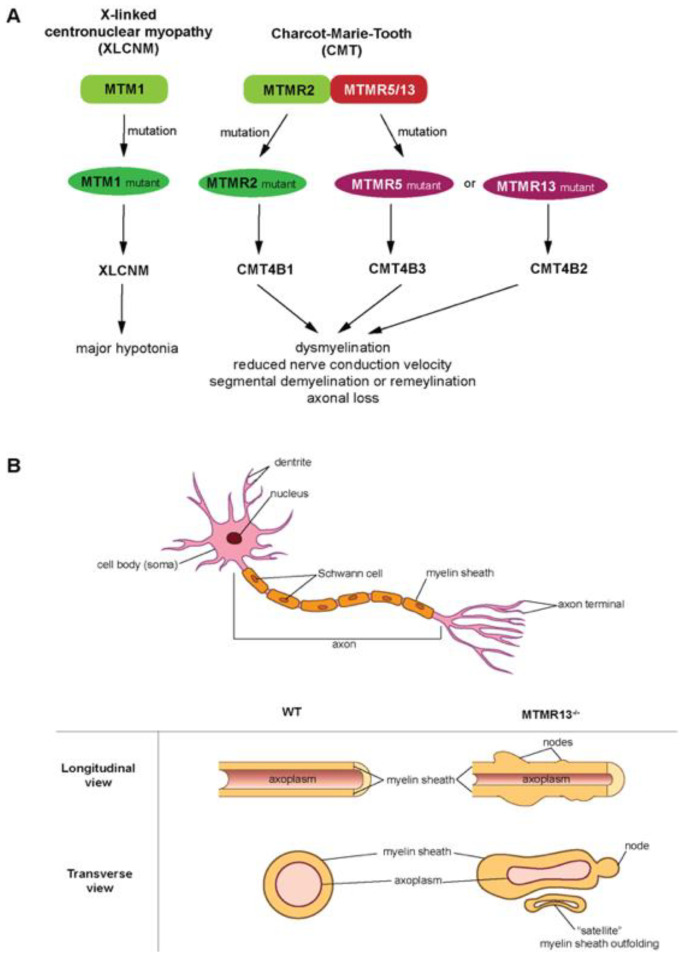
Myotubularins (MTMs) in diseases: (**A**) X-linked centronuclear myopathy (XLCNM) and Charcot-Marie-Tooth (CMT), pathologies associated with MTM family mutations. XLCNM is a rare congenital myopathy characterized by decreased muscle tone or weakness (hypotonia), and is caused by mutations to the *MTM1* gene. CMT denotes a group of peripheral nerve neuropathies with an incidence of ~1 in 2500 people, and is characterized by abnormal myelin folding, diminished nerve conduction velocities, as well as peripheral axon loss. CMT type 4B (CMT4B) is caused by mutations in the MTM family proteins (MTMR2, MTMR5 or MTMR13) which lead to CMT4B1, CMT4B3 or CMT4B2, respectively. (**B**) (Top) Canonical representation of a PNS neuron and its various processes. (Bottom) Idealized illustration adopted from Robinson et al. [30] of the aberrant myelin phenotypes associated with CMT4B2 (longitudinal and transverse views of MTMR13-/- knockout axons) as compared to Wild Type (WT) axons. Abnormal myelination, as depicted by “satellite” myelin outfoldings, as well as myelin “nodes”, are present in mouse models deficient of MTMR13, resulting in diminished nerve conduction velocities typical of CMT4B patients.

Understanding the MTMR2-MTMR13 heterodimer as a signal regulator will provide insight useful for developing drugs towards these proteins. For example, mutations of MTMR2 and MTMR13 leads to misregulation of the AKT signaling in Type 4B Charcot-Marie-Tooth (CMT4B) disease [76]. In particular, these mutations affect the phosphatidylinositol (PtdIns) dynamics (Figure 1B). The active MTMR2 dephosphorylates PtdIns3P and PtdIns(3,5)P_2_, thereby regulating membrane trafficking [76,77]. Overexpression of MTMR2 prevents sustained activation of epidermal growth factor, which leads to sustained AKT activation in transgenic mice [76]. The MTMR13 pseudophosphatase counteracts this blockage of epidermal growth factor [76]. The MTMR2-MTMR13 complex’s role in AKT signaling solidifies this complex regulation of phosphoinositide-regulated cellular processes. PtdIns(3)P and PtdIns(3,5)P_2_ are important molecules for endosomal-lysosomal membrane trafficking [70], which is tightly regulated by PtdIns kinase and phosphatase activity. The phosphatase-pseudophosphatase MTM complex selectively hydrolyzes the 3-carbon phosphate group on either PtdIns(3)P or PtdIns(3,5)P_2_ through a conserved Cys-X_5_-Arg motif (Figure 1B) [76]. The active MTMR2 may dimerize with inactive MTMR5/Sbf1, MTMR9, MTMR12/3-PAP, or MTMR-10 (Figure 1) to stabilize a complex with PtdIns phosphatases (Figure 1B), as well as active MTMR1 [72]. These various interactors of MTMR2 highlight the complexity of its signaling network, as each interactor interacts with other MTMs (Figure 1A). MTMs have also been linked to X-linked centronuclear myopathy (XLCNM) (Figure 2A) [78].

Mutants of another pseudophosphatase myotubularin 1 (MTM1), distinctively different from MTMR1, lead to XLCNM [78]. MTM1 forms homodimers, as well as heterodimers with MTMR10 or MTMR11 (pseudophosphatses) [72,78]. Furthermore, heterodimers are linked to several types of CMT4, CMT4B1, CMT4B3, and CMT4B2 (Figure 2A). CMT4B is the predominant; mutations in MTMR2, MTMR13 and MTMR5 result in CMT4B1/2/3, respectively [79,80,81,82]. MTMR2 (phosphatase) forms heterodimers with MTMR13 (pseudophosphatase) or MTMR5 (pseudophosphatase), and heterodimers with MTMR13 (pseudophosphatase) or MTMR5 (pseudophosphatase), and disruption of these dimers leads to the associated CMT pathology [83]. This linkage to CMTs suggests myotubularins have a role in neuronal signaling (Figure 2). Genetic studies in mice revealed that MTMR12 interacts with another phosphatase, polyphosphoinositde phosphatase (FIG4) (Figure 1B), and knockout of these genes resulted in the myelin outfolding (Figure 2B) [76]. The active enzyme (MTMR2) binds the pseudophosphatase MTMR13 (Figure 1A and Figure 2A) [22]. A mutation in the MTMR13 pseudophosphatase, or the MTM2 active homolog of the complex, as well as disassociation of the complex, can lead to diseases [30,71] such as Type 4B CMT [69,84] and axon degeneration [71,84]. This demonstrates the functional importance of the pseudophosphatase component in the interaction between MTMR2 and MTMR13. In addition, the MTMR2 and MTMR13 complex is also impacted in glaucoma [85] and cancer [86,87], and both MTMR2 and MTMR13 are referred to as disease-causing proteins [76]. MTM pseudophosphatase:phosphatase heterodimer signaling complexes are required for cellular processes such as differentiation, membrane trafficking, endocytosis, and survival [10,27,69,70]. Furthermore, MTM heterodimer complexes are essential for proper MTM function [27,71].

## 3. Tensin in Disease

**Figure 3:**
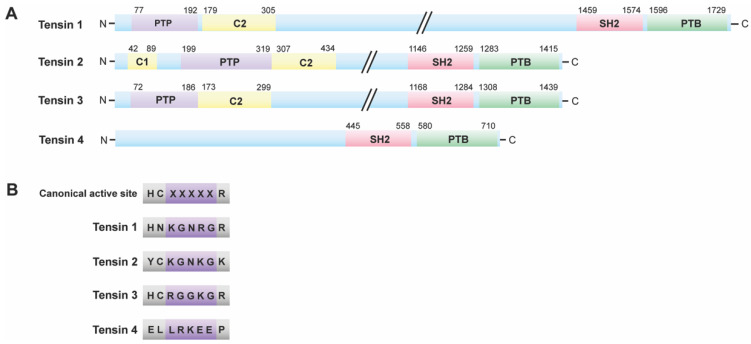
Tensin family: (**A**) Structure of tensin family members. All tensin family members have a C-terminal PTB (phosphotyrosine-binding) domain preceded by an SH2 (Src 2Homology 2) domain. These structures vary beyond the SH2 and PTB domains. (**B**) Active sites of tensin family members. Some tensin family members have active site motifs that differ from the catalytically active canonical PTP HCX_5_R motif. Tensin 1 and 2 are the pseudophosphatase members of the family, whereas tensin 3 possibly retains catalytic competence. Tensin 4 lacks a PTP domain; therefore, it is neither a phosphatase nor a pseudophosphatase.

MTMs are not the only PTPs that dephosphorylate lipids. Phosphatase and tensin homologue (PTEN) is a dual specificity phosphatase that functions as a protein and lipid phosphatase [88]. PTEN dephosphorylates the phosphatidylinositol (3,4,5)-trisphosphatase (PIP3), a second messenger that activates protein kinase B (PKB/AKT) [88,89]. PTEN is a well known tumor suppressor, and its function in cell survival is very well established [90,91,92,93]. However, the function of its inactive homolog, tensin, is not as thoroughly investigated. Tensin is a family of multi-domain scaffold proteins that consists of tensin 1, tensin 2, tensin 3, and C-terminus tensin (c-ten) or tensin 4 (Figure 3A) [64,66].

All paralogs have a C-terminal phosphotyrosine-binding (PTB) domain preceded by a Src 2 homology (SH2) domain (Figure 3A), which also allows it to bind to phosphotyrosine residues [64]. In addition, they possess an N-terminal PTP domain followed by a protein kinase C conserved region 2 (C2) domain (membrane-localizing unit), with the exception of tensin 4, which lacks both the PTP and C2 domains [64]. Along with this C2 domain, tensin 2 has another membrane-localizing functional unit, an N-terminal protein kinase C conserved region 1 (C1) (Figure 3A) [64], indicating its localization to the membrane. Within the PTP active site of tensins, the catalytically active canonical HCX_5_R motif differs (Figure 3B). Tensins 1 and 2 have mutations Cys to Asp and His to Tyr, respectively, that categorizes them as pseudophosphatases (Figure 3B) [64], based on sequence analysis. Furthermore, tensin 4 does not have a PTP domain (Figure 3), auspiciously placing it as a noncatalytic molecule. Therefore, tensin 4 is not a phosphatase or pseudophosphatase. However, tensin 3 retains the signature active motif (Figure 3B), thus maintaining catalytic competence, and the ability to dephosphorylate residues.

**Figure 4:**
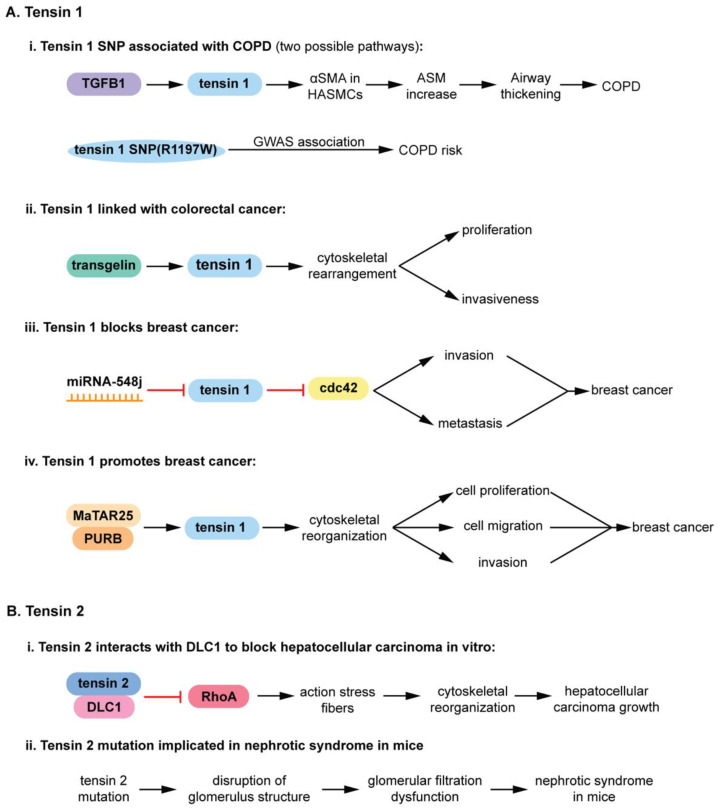
Roles of tensin family members in disease: (**A**) Tensin 1 roles in disease. (**i**) Tensin 1 SNP is linked to chronic obstructive pulmonary disease (COPD) thorough experimental evidence and GWAS. TGFβ1 upregulates tensin 1 (R1197W) expression, increasing downstream effectors leading to airway thickening that is characteristic of COPD. (**ii**) Tensin 1 is linked with human colorectal cancer through transgelin signaling, upregulating metastatic and proliferative signaling of colorectal cancer cells. (**iii**) Tensin 1. Tensin 1 translation is prevented by miRNA-548j, resulting in hyperactivation of cdc42 that increases invasion and metastasis in breast cancer. (**iv**) Tensin 1 promotes breast cancer. The MaTAR25 lncRNA interacts with PURB to increase tensin 1 mRNA and protein levels, resulting in cytoskeletal rearrangements that augment proliferation, migration, and invasion in breast cancer cells. (**B**) Tensin 2 roles in disease. (**i**) Tensin 2-DLC1 (deleted in liver cancer 1) complex inhibits RhoA. This prevents RhoA-mediated actin stress fiber generation, which in turn prevents cytoskeletal rearrangements that support hepatocellular carcinoma growth. (**ii**) Tensin 2 mutation implicated in nephrotic syndrome in mice. An 8-nucleotide deletion in tensin 2 triggers a frame shift that introduces a nonsense mutation. This mutant gene is associated with lower tensin 2 mRNA and protein levels in mouse kidneys. These tensin 2 deficient mice present with disruption of glomerulus structure and glomerular filtration dysfunction, suggesting that tensin 2 deficiency leads to nephrotic syndrome in an animal disease model.

Since tensin 1 and tensin 2 are pseudophosphatases, they will be described in more detail. Knockout mouse studies have linked their functionality to muscle regeneration and to diseases (Figure 4) such as kidney failure, cancer, chronic obstructive pulmonary disease (COPD), etc., [31,64,66,68,94,95]. Tensin 1 is a 220-kD cytoplasmic phosphoprotein localized to focal adhesions, serving as a molecular bridge linking the extracellular matrix (ECM), actin cytoskeleton, and signal transduction [64,66,95,96]. A tensin 1 single nucleotide polymorphism (SNP) at position 1197 from an arginine to a tryptophan (R1197W) correlates with an increase in COPD. TGFβ1 upregulates the expression of mutated (R1197W) tensin 1, increasing α smooth muscle actin (αSMA) expression (Figure 4(Ai)) [32]. αSMA is a major component of human airway smooth muscle cells (HASMCs). Therefore, an increase in αSMA expression increases airway smooth muscle (ASM), possibly contributing to the characteristic airway thickening of COPD (Figure 4(Ai)) [32]. Tensin 1 is also linked to human colorectal cancer (Figure 4(Aii)).

Transgelin (actin binding protein) enhances expression of tensin 1, which triggers cytoskeletal rearrangements and leads to proliferation and metastasis of colorectal cancer cells (Figure 4(Aii)) [66]. Moreover, poor survival of patients with colorectal cancer has been associated with elevated levels of transgelin and tensin 1; siRNA of both these proteins decreases proliferation and invasion [66]. Recently, tensin 1 has been linked to breast cancer (Figure 4A). Intriguingly, tensin 1 appears to have paradoxical roles in decreasing breast cancer (Figure 4(Aiii)) [68] as well as promoting breast cancer (Figure 4(Aiv)) [68]. As an inhibitor, tensin prevents the Rho GTPase cell division cycle 42 (cdc42) from performing its duties of signaling cells to become invasive and metastasize [68]. Cells expressing the microRNA (miRNA) 548j prevents the translation of tensin 1, which inhibits the RhoGTPAse cell division cycle 42 (cdc42) [67]. The hyperactivated cdc2 promotes invasion and metastasis in breast cancer (Figure 4(Aiii)). An elevated expression of tensin 1 also promotes breast cancer (Figure 4(Aiv)) [68], highlighting the importance of a balanced expression of this pseudophosphatase. The mammary tumor associated RNA 25 (MaTAR25) long non-coding RNA (lncRNA) interacts with purine rich element binding protein B (PURB), and increases the expression of tensin 1, mRNA and protein levels [68], thereby contributing to cytoskeletal rearrangements that augment proliferation, migration, and invasion in breast cancer cells (Figure 4(Aiv)) [68]. The various dynamics of tensin 1 and its upstream and downstream signaling molecules in diseases (Figure 4A) suggests a strong potential that this pseudophosphatase may serve as therapeutic targets for diseases [64].

Tensin 2 is also a pseudophosphatase that has a critical role in diseases such as cancer and renal failure (Figure 4B) [64]. It is a 152 kDa focal adhesion protein with high homology to tensin 1 that localizes at the end of stress fibers [97]. Similar to tensin 1, tensin 2 also has an inhibitory effect by interacting with the tumor suppressor gene, deleted in liver cancer (DLC1), and inhibiting Rho GTPase-activating protein (RhoGAP) (Figure (4Bi)) [94]. DLC1 encodes three domains, Rho GTPase-activating protein (RhoGAP), and steroidogenic acute regulatory-related lipid transfer [94]. Tensin 2 was the first protein identified to interact with DLC1, which it does through its PTB domain [94]. In addition, both DLC1 and tensin 2 contain a caveolae binding motif, and the tensin-2-DLC1 complex interacts with caveolin-1 [94]. Tensin-2-DLC1 complex colocalizes to the caveolae in hepatocellular carcinoma (HCC), and inhibits cellular growth [94]. Tensin 2 also has a role in the nephrotic syndrome (a kidney disorder in which too much protein is released proteinuria) (Figure 4(B2ii)) [31]. A congenital nephrotic syndrome mouse model (ICGN mice) have an eight-nucleotide deletion in tensin 2, which triggers a frame shift that introduces a nonsense mutation [31]. This demonstrates the critical role of tensin 2 in regulating proper kidney function. ICGN is a mouse strain with hereditary nephrotic syndrome, from which affected mice have damaged podocytes (kidney cells wrapping around glomerular capillaries), albuminuria (too much albumin in urine), and edema (swelling of tissues caused by fluid) [31]. Tensin 2 is deleted in these mice [31], establishing the critical role of tensin 2 in normal kidney function. In addition, the mice that lack tensin 2 have disruption of glomeruli (capillaries near the end of kidney tubules where waste is filtered), resulting in glomerular filtration dysfunction [31]. Tensin 2 deficiency in ICGN mice leading to the nephrotic syndrome provides strong evidence that this pseudophosphatase may be an important target for renal failure. Taken together with the role of tensin 1 in diseases, it is apparent that the pseudophosphatase members of the tensin family may serve as targets for therapeutic interventions in renal disease, cancers, breast, liver, etc., and COPD [64]. Furthermore, elevated tensin 1 expression is a potential biomarker for colorectal cancer [66], further solidifying pseudophosphatases as therapeutic targets.

## 4. STYX Pseudophosphatases in Disease

**Figure 5:**
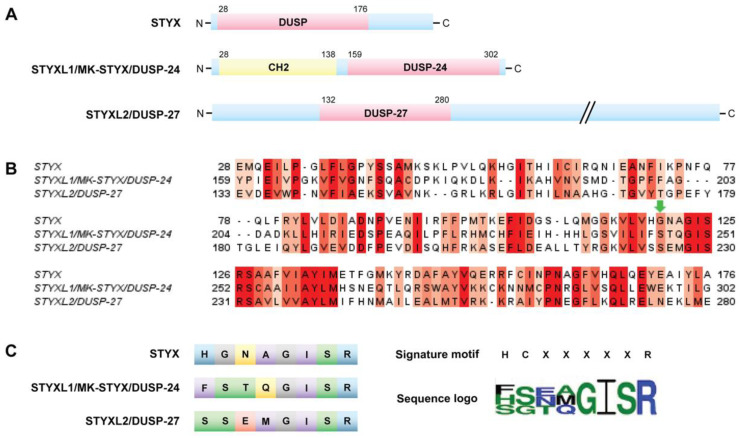
STYX pseudophosphatases: (**A**) Structure of STYX pseudophosphatases. STYX has an N-terminal DUSP domain. MK-STYX features an N-terminal CH2 domain and a C-terminal DUSP domain. STYXL2 contains an N-terminal DUSP domain. (**B**) Sequence alignment of the phosphatase domains of the STYX pseudophosphatases (Clustal Omega 1.2.4). Conserved residues are shown in red, where darker red indicates higher conservation (Jalview 2.11.14 with a threshold of >5.5). The green arrow indicates where the essential active-site cysteine residue would be located in a catalytically active PTP (HCX_5_R). (**C**) The left-hand side of the panel compares the active site sequences of the STYX pseudophosphatases. Amino acid color indicates chemical property. Polar amino acids are shown in green, neutral in yellow, basic in blue, acidic in red, hydrophobic in purple, and glycine in grey. The right-hand side of the panel shows the sequence of the consensus active signature motif of PTPs compared to the sequence logo of the three STYX pseudophosphatases. The sequence logo was built by WebLogo 3.7.4 with a 2.0-bit scale.

The term “STYX” (phosphoserine/threonine/tyrosine-interacting protein) was coined to designate the phosphotyrosine binding domain that has no catalytic activity, or “dead” phosphatases or pseudophosphatase [12]. These “dead” or STYX domain phosphatases allude to the Greek mythological STYX river of the dead [11,16]. Usage of a point mutation to “restore” catalytic activity in the STYX domain has proved to be a helpful tool to initiate the process of investigating molecules that contain STYX domains. STYX domain phosphatases are pseudophosphatases; therefore, the authors have referred to them as STYX pseudophosphatases, as previously reported [11]. To date, there are three STYX pseudophosphatases: STYX, STYX-like-1 (STYXL1), and STYX-like-2 (STYXL2) (Figure 5).

To date, there are three STYX pseudophosphatases: STYX, STYX-like-1 (STYXL1), and STYX-like-2 (STYXL2) (Figure 5). They are all members of the dual specificity protein tyrosine phosphatase (DUSP) family; however, they belong to different subfamilies [5]. In fact, the DUSP STYX domain is their only commonality (Figure 5), in which STYXL1 and STYL2 have a DUSP nomenclature, DUSP-24 [6,13,17] and DUSP-27 (Q5VZP5, Uniprot) [5,7], respectively. These domains vary both from the active site signature motif and each other (Figure 5B,C). Of note, all these STYX domain proteins have glycine, isoleucine, serine, and arginine in the active site motif (Figure 5B,C). Furthermore, early reports classified glycine as a conserved residue of the active site motif, HCxxGxxR [10,24]. STYXL1 was the second STYX domain protein characterized and, therefore, was appropriately assigned as STYX-like-1 (STYXL1) protein [11]. Because STYXL1 is the inactive member of the MAP kinase phosphatase (MKP) subfamily [11,13,17], it is also referred to as MK-STYX, the preference of these authors. However, the gene nomenclature is *STYXL1*.

**Figure 6:**
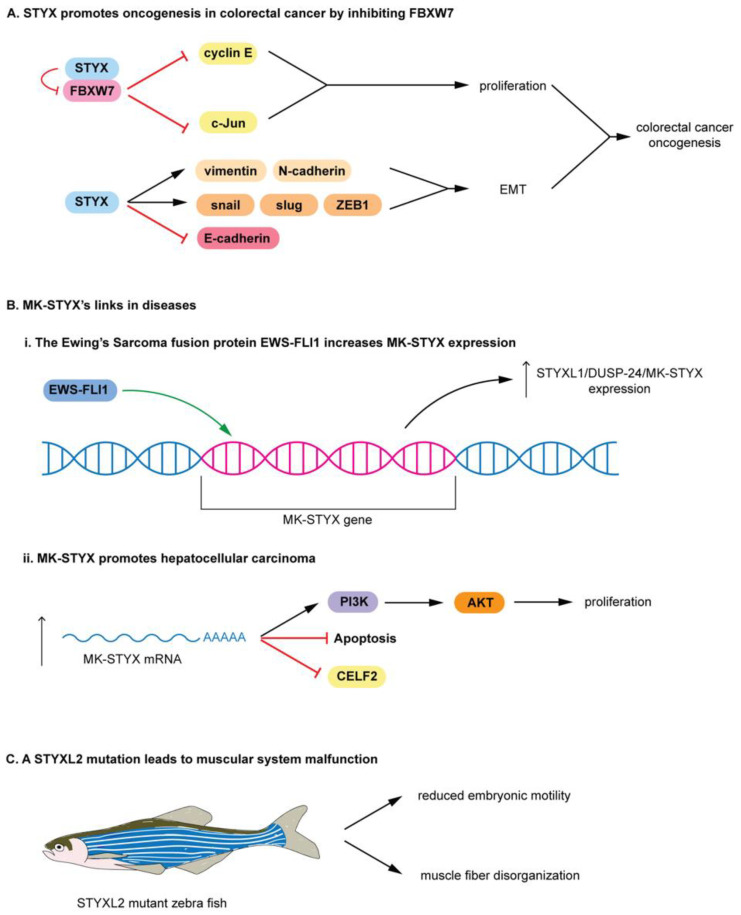
Roles of STYX domain pseudophosphatases in disease: (**A**) Prototypical STYX roles in colorectal cancer. STYX promotes oncogenesis in colorectal cancer by inhibiting FBXW7. STYX binds FBXW7, preventing other interactions of FBXW7, which is a substrate recruiter for a ubiquitin protein ligase complex. Thus, STYX prevents the degradation of cyclin E and c-Jun, promoting proliferation in colorectal cancer. STYX overexpression increases the expression of vimentin, N-cadherin, snail, slug, and ZEB1, but reduction in E-cadherin. These proteins support EMT; STYX may promote the oncogenesis of colorectal cancer by positively regulating EMT. (**B**) MK-STYX’s links in diseases. (**i**) The Ewing’s sarcoma fusion protein EWS-FLI1 increases MK-STYX expression. The EWS-FLI1 oncoprotein binds an ETS binding motif within the MK-STYX gene and increases the MK-STYX’s expression. (**ii**) MK-STYX promotes hepatocellular carcinoma. An increase in MK-STYX mRNA expression leads to upregulation of PI3K/AKT pathway proteins and an enhancement of proliferation in hepatocellular carcinoma, while inhibiting apoptosis and CELF2. (**C**) STYXL2 mutation leads to muscular system malfunction. A transgene integration into the STYXL2 gene reduces STYXL2 expression in zebrafish, resulting in reduced embryonic motility, low spontaneous coiling movements, and severely reduced touch response, as well as major muscle fibers disorganization.

The nomenclature for pseudophosphatase STYX-like-2 (STYXL2) is not as straightforward. It is the first protein designated as DUSP-27 [98], but shares its name with a DUSP-27 active phosphatase, which is a very different protein [5,99]. Therefore, it is challenging to determine its actual activity, which remains to be determined [5], and it has been suggested that it be referred to as STYXL2 by Friedberg et al. [98]. Nevertheless, these STYX domain proteins are classified as pseudophosphatases. STYX and MK-STYX have been shown to be important signaling regulators in cellular processes such as cell cycle, spermatogenesis, cell-fate decisions, cell migration, ubiquitylation and protein degradation, apoptosis, and neuronal differentiation [25,26,51,52,54,100,101], alluding to their roles in the etiology of various diseases. Furthermore, these STYX pseudophosphatases have roles in cancer such as colorectal, Ewing sarcoma, and hepatocarcinoma [8,28,33]. The first catalytically inactive DUSP characterized is STYX [8]. STYX has a glycine residue in place of the essential active-site cysteine residue (Figure 5B,C) [12]; a single point mutation of glycine to cysteine restores its catalytic activity [12]. STYX inhibits differentiation as modulator of cell-fate decisions and cell migration through spatiotemporal regulation of ERK1/2 signaling, by competing with DUSP4 (MKP-2) for binding to ERK1/2 [7]. In addition, STYX disrupts the morphology of the Golgi apparatus in an ERK-dependent manner, affecting directional cell migration. STYX regulates ubiquitylation by regulating the ubiquitin ligase SKP/CUL1-F-box (SCF) complex [4,5,47]. STYX interacts with the F-box protein WD40 (tryptophan and aspartic acid repeats) domain, FBXW7, a substrate recruiter for a ubiquitin protein ligase complex [47], and a tumor suppressor [4,5,102,103,104], and inhibits degradation of substrates [47]. Furthermore, the expression of STYX and FBP is implicated in breast cancer patients, where patients’ survival is modulated by the disruption of the balance between STYX and FBXW7 expression [47]. In addition, STYX promotes oncogenesis in colorectal cancer by inhibiting FBXW7, blocking the degradation of cyclin E and c-Jun (Figure 6A) [28]. Therefore, free (unbound) cyclin E and c-Jun are able to promote proliferation in colorectal cancer. STYX overexpression increases vimentin, N-cadherin, snail, slug, and ZEB1, but reduces E-cadherin (Figure 6A). Because these proteins support epithelial to mesenchymal transition, STYX may promote the oncogenesis of colorectal cancer by positively regulating the epithelial to mesenchymal transition.

MK-STYX is also linked to cancers. MK-STYX lacks the critical cysteine in the active site signature motif (HCX_5_R) (Figure 5) [4,6,11,13,105], resulting in it being catalytically inactive [4,6,11,13,105]. It has a phenylalanine and serine in this signature motif (Figure 5), replacing histidine and the essential cysteine, respectively. It also has a presumed kinase interaction motif (KIM) [4,11,13], which binds MAPK/ERK1/2 [106]. However, MK-STYX has mutations within the KIM domains. It is missing critical arginine residues required for MAPK/ERK docking [6,107,108]. Although these mutations may render the phosphatase catalytically inactive, MK-STYX, is a bona fide signal regulator of many cellular processes, such as translation, stress response, apoptosis, and neuronal differentiation [6,13,51,52,53,55,109]. Furthermore, the misregulation of MK-STYX (*STYXL1*) has been found in Ewing’s sarcomas (Figure (6Bi)) [4,7,8,9]. The Ewing’s sarcoma friend integration leukemia 1 fusion protein (EWS-FLI1) increases MK-STYX expression. EWS-FLI1 oncoprotein binds an ETS binding motif of *STYXL1*, greatly increasing the expression of MK-STYX (Figure 6Bi), resulting in Ewing sarcoma [8], a pediatric bone cancer. An increase in MK-STYX expression also promotes hepatocellular carcinoma (Figure 6(Bii)) [33]. Increased MK-STYX mRNA expression results in upregulation of PI3K (phosphatidyl 3-kinase)/AKT pathway proteins and an enhancement of proliferation, while reducing apoptosis in hepatocellular carcinoma (Figure 6Bii) [33]. In addition, MK-STYX downregulates CUGBP, elav-like family member 2 (CELF2) (Figure 6Bii) [33], which may serve as the rationale for the pro-proliferative and anti-apoptotic functions of MK-STYX in hepatocellular carcinoma. The relationship of STYXL2 (DUSP-27) to diseases is not as well established as that of STYX and MK-STYX; however, STYXL2 may have a potential role in paralysis [59,99]. STYXL2 lacks the critical conserved cysteine reside, which was replaced by serine (Figure 5B,C) [59], and is expressed in skeletal and cardiac muscle [99]. STYXL2 is a downstream target of the Janus activated kinase/Signal transducers and activators of transcription signaling pathway [99], suggesting that STYXL2 has important roles in signaling cascades. For example, knockdown of STYXL2 inhibits myogenic differentiation [5,99].

In addition, a transgene integration into *STYXL2* reduces STYXL2 expression in zebrafish, resulting in muscular system malfunction (Figure 6C) [5,99]. STYXL2 mutants have reduced embryonic motility (low paralysis), displaying low spontaneous coiling movements and severely reduced touch response (Figure 6C) [99]. Furthermore, STYXL2 mutants have a major disruptions in the contractile apparatus of their muscle fibers–disorganized muscle fiber formation (Figure 6C) [99].

## 5. Atypical Pseudophosphatases

This review focused on the less contradictory pseudophosphatases’ signaling in diseases, with DUSP27/STYXL2 as the exception; Reiterer et al. encompasses such contradictory pseudophosphatases [5]. In addition, the D2 domains, which are catalytically inactive and recognize substrate [10], of the PTP receptor molecules were excluded. D2 is a tandem domain of the active PTPs that has mutations that make it an inactive DUSP domain [10,110]. These D2 domains may be significant; however, we focused on the current literature of the pseudophosphatases. Recently, the protein tyrosine phosphatase receptor U (PTPRU) was classified as pseudophosphatase [111], both its DUSP domains, D1 and D2, were considered as pseudophosphatase domains [111]. However, PTPRU was reported to display catalytic activity, although it consists of the canonical HCX_5_R active motif [112,113]. PTPRU inhibits lung cancer [114] and may promote glioma [115], both by dephosphorylation of β catenin [114,115]. Recently, it has been considered a tumor suppressor, as it inhibits cancer stemness through attenuating the hippo/YAP signaling [29]. In addition, tensin 2 is thought to have possible activity by dephosphorylating insulin receptor substrate-1 [116]. Thus, the relevance of the contextual situations such as signaling context, cell type, etc., is more pronounced and necessary to understand the complexity of their functions [4,5].

Some pseudophosphatases were reported as having catalytic activity [4,5,7]. Similarly, some active PTPs display pseudophosphatase activity that is critical for their role as regulators in cellular processes [5]. Phosphatase of regenerating liver-3 (PRL3) phosphatase is an example of the concept of a pseudo-pseudophosphatase, which recognizes the catalytic activity and the importance of the noncatalysis in signaling cascades [5,117,118]. PRL3 pseudophosphatase activity promotes metastatic growth [118]. In studies with PRL3 mutants specifically defective in either binding the substrate of PRL3 substrate, CBS domain divalent metal cations transport mediators (CNNM) or phosphatase activity [118] demonstrated that the phosphatase activity is dispensable [118]. Moreover, phosphatase activity prevents PRL3-CNNM interaction, which is necessary and sufficient for tumor metastasis [118]. The concept of active phosphatases functioning as pseudophosphatases may help guide the design of inhibitory therapeutics. However, it is very important to not confuse a phosphatase with a pseudophosphatase. If it has a catalytic domain and dephosphorylates substrates, it is a phosphatase, not a pseudophosphatase.

## 6. Conclusions

The origin of pseudophosphatases was through bioinformatics and sequence analysis [1,5,11,12,17,119]. Sequence alignment demonstrated mutations of critical residues that were perceived to render an enzyme inactive [1,2,119], “birthing” the pseudoenzyme discipline. Initial studies from Jack Dixon’s group provided a clear example of using bioinformatics to define pseudophosphatases. They showed that resubstituting the critical cysteine residue back in the active signature motif of STYX restores activity [12]. This was also demonstrated with MK-STYX [13]. Perhaps, defining STYX and MK-STYX bioinformatically as pseudophosphatase held true, because cysteine, which is missing in both proteins, is the critical residue for dephosphorylation [10]. The caveat is that not all mutations of the canonical enzyme leads to impaired enzymatic activity. Simply using bioinformatics is not enough, and is making the field much more complicated. Therefore, it is important for the field to come to the consensus and/or extend how pseudophosphatases are defined. Classifying an active phosphatase, regardless of whether it has minimal or robust activity, as a pseudophosphatase is confusing and could potentially be detrimental in recruiting new, young, and talented researchers to this discipline. A collective focus towards combinational approaches to investigate these molecules within their actual contextual environment, and the appropriate substrates or binding partners, will significantly advance the field.

It is apparent that STYX domain proteins are essential signaling regulators. They are pivotal in demonstrating that pseudophosphatases and pseudoenzymes are able to maintain a three-dimensional fold as well as having the ability to bind phosphorylated proteins [4,5,6,11,16]. Moreover, by demonstrating that these catalytically inactive proteins serve functionalities that are essential in many diseases, their worthiness for strong consideration of therapeutic drug targets has been indicated.

The steadfastness of researchers investigating the pseudoenzyme field continues to provide the foundation of these catalytically inactive enzymes as critical signaling regulators. While pseudophosphatases may serve as dominant negative antagonists (competitors) of endogenous protein phosphatases [4,11,16,54], the field has advanced its knowledge, demonstrating that these proteins also serve as anchors, integrators, and modulators in cellular processes [5,7]. Furthermore, the interest of new investigators, collaborative efforts, and the development of new technologies have demonstrated their role in diseases. Should designing drugs focus on catalytic active (phosphatase) or noncatalytic function?

The field has established itself, highlighting pseudophosphatases as critical selective regulators of signaling cascades, building the foundation for pseudophosphatases’ roles in pathologies, resulting in the focus of pseudophosphatases in diseases such as neuropathies (CMT), various cancers, and COPD, setting the platform to consider pseudophosphates as therapeutic targets. This special edition of protein tyrosine phopsphatases as therapeutic targets emphasizes that PTPs are druggable. Furthermore, the inclusion of inactive members of PTPs, pseudophosphatases is timely and warranted. This review detailed substantial and compelling evidence that pseudophosphatases are critical players in diseases. Of course, there is much remaining work to advance the field further. With each decade we are advancing the field. The implication of pseudophosphatases as important molecules in diseases solidifies our belief that these proteins are suitable for drug targets. Thus, it is important to provide a platform to consider them as therapeutic targets. An important aspect of this platform will be the development of new tools and techniques to investigate pseudophosphatases. Proteomics and biochemical approaches will continue to be instrumental in understanding the molecular mechanisms of pseudophosphases [4,51,109], while structural biology, biochemistry, and in vivo (transgenic animals) models must be used to elucidate the molecular mechanisms and biological functions of these pseudoenzymes [4,5,6,119]. Understanding the structure-function relationship of pseudophosphatases may serve as the blueprint for designing therapeutics for these pseudophosphatases [4,5]. In addition, the combination of computational studies to analyze these proteins will provide a more robust characterization of these pseudoenzymes and/or pseudophosphatases [5,6].

It is an exciting time to investigate pseudophosphatases, especially with many new technologies and newcomers mapping out their roles in diseases. This, in combination with elucidating the details of their mechanistic modes of action, will provide the foundational platform required to successfully investigate pseudophosphatases as therapeutic drug targets. It is exciting to have contributed to this field since its infancy and observe pseudophosphatases emerge into recognition as essential regulators of signaling cascades, their increased implications in diseases, and now as strong potential therapeutic targets.

## Figures and Tables

**Table 1:** Pseudophosphatase Functions and Roles in Disease.

Pseudophosphatase Name	Alternative Names	Normal Function	Role in Disease
MTMR5 (Myotubularin-related protein 5)	SBF1	Heterodimerizes with and increases phosphatase activity of MTMR2 [34]	Mutations in MTMR5 cause CMT4B3 [35] Male mice with MTMR5 knockout are infertile [36]
MTMR9 (Myotubularin-related protein 9)		Heterodimerizes with and increases phosphatase activity of MTMR6, MTMR7, and MTMR8 [37] Negative regulator of autophagy [37] Negative regulator of apoptosis [37]	An MTMR9 SNP is positively associated with obesity [38] Mutated MTMR9 is implicated in epilepsy [39]
MTMR10 (Myotubularin-related protein 10)		-	MTMR10 expression decreased in the esophageal mucosa of sufferers of esophageal achalasia [40]
MTMR11 (Myotubularin-related protein 11)		-	The expression of MTMR11 is reduced in acute myeloid leukemia [41] Abnormal expression levels in HER2 breast cancer cells [42]
MTMR12 (Myotubularin-related protein 12)	3-PAP	Dimerizes with and stabilizes MTM1 [43] Dimerizes with MTMR2 [44]	-
MTMR13 (Myotubularin-related protein 13)	SBF2	Heterodimerizes with and increases phosphatase activity of MTMR2 [30]	Mutations in MTMR13 are the causative factor of CMT4B2 [45]
STYX (Serine/threonine/tyrosine -interacting protein)		Major positive regulator of spermatogenesis. Interacts with CRHSP-24 [12] Anchors ERK1/2 in the nucleus and reduces its phosphorylation [46] Binds to FBXW7 and reduces its association with the SCF ubiquitin ligase complex [47]	Drives tumor growth and metastasis in colorectal cancer [28] Expression increased in breast cancer [48] Downregulates FBXW7 to drive endometrial cancer [49]
MK-STYX (Mitogen-activated protein kinase phosphoserine/ threonine/tyrosine-binding protein)	STYXL1, DUSP-24	Antagonizes stress granules, and interacts with G3BP1 [13,50] Promotes mitochondrial dependent apoptosis by reducing the activity of PTPMT1 [51] Promotes cellular changes associated with neuronal differentiation [52,53,54] Alters nuclear localization of HDAC6, and increases the detyrosination of tubulin [55]	Overexpressed in Ewing’s sarcoma [9] Commonly amplified in glioma and enhances glioma growth, migration, and metastasis [56] MK-STYX expression elevated in hepatocellular carcinoma (HCC). Has pro-proliferative and anti-apoptotic effects on this cancer. [33] Expression increased in breast cancer cells subjected to proton beam therapy [57] MK-STYX expression is elevated in human prostate cancer [58]
STYXL2 (Serine/threonine/tyrosine-interacting-like protein 2)	DUSP-27 (duplicated)	Expressed in muscle tissue and may be important for myofiber development [59]	Mutation in STYXL2 causes muscular dysfunction [59]
TAB1 (TGF-beta-activated kinase 1 and MAP3K7-binding protein 1)	MAP3K7IP1	Activates TAK1, a MAP3K, during TGFβ signaling [60] TAB1 activates p38 MAPK and changes its localization [61] TAB1 blocks MDM2′s inhibition of p53 [62]	Cleaved by the Enterovirus 71 virus that causes hand, food, and mouth disease (HFMD) [63] TAB1 levels reduced in ovarian cancer [62]
Tensin 1	TNS1	Serves as an adaptor between integrin receptors and actin filaments in focal adhesions [64] Upregulates cell migration [65]	A common tensin 1 SNP is positively associated with COPD [32] Increased expression augments proliferation and metastasis in colorectal cancer [66] Tensin 1 inhibits cdc42 to reduce invasion and metastasis in breast cancer [67] A MaTAR25/PURB complex activates tensin 1 to drive breast cancer [68]

The proteins here all contain alterations in their catalytic active site motifs that are associated with loss of phosphatase function.

## Abbreviations

ASM	airway smooth muscle
αSMA	α smooth muscle actin
C1	protein kinase C conserved region 1
C2	protein kinase C conserved region 2
CNNM	CBS domain divalent metal cations transport mediators
cdc42	cell division cycle 42
CELF2	CUGBP, elav-like family member 2
D1	domain 1 of PTP
D2	domain 2 of PTP
ERK	extracellular signal-regulated kinases 1/2
ETS	E-twenty-six transformation-specific
CH2	cell division cycle 25 phosphatase homology 2
c-ten	C-terminus tensin
CMT	Charcot-Marie-Tooth
COPD	chronic obstructive pulmonary disease
DLC1	deleted in liver cancer 1
DUSPs	dual specificity phosphatases
DMB	dynein binding domain
EMT	epithelial to mesenchymal transition
EWS-FLI1	Ewing’s sarcoma and friend leukemia integration 1 fusion protein
FBXW7	F-box protein WD40 (tryptophan and aspartic acid repeats) domain
HASMCs	human airway smooth muscle cells
HD-PTP	histidine domain containing protein tyrosine phosphatase
KIM	kinase interaction motif
MaTAR25	mammary tumor associated RNA 25
MAP	mitogen-activated protein kinase
MKP	MAP kinase phosphatase
MK-STYX	mitogen-activated protein kinase phosphoserine/threonine/tyrosine-binding protein
MTMs	myotubularin phosphatases
MTM1	myotubularin 1
MTMR#	myotubularin- related protein with assigned number
PIP_3_	phosphatidylinositol (3.4.5)-trisphosphate
PKB/AKT	protein kinase B
PRL3	phosphatase of regenerating liver- 3
PtdIns	phosphatidylinositol
PNS	peripheral nervous system
PTEN	phosphatase and tensin homologue
PTPM1	PTP localized to the mitochondrion 1
PTP	protein tyrosine phosphatase
PURB	purine rich element binding protein 1
RhoGAP	Rho GTPase-activating protein
SBF2	Sbf2 = SET binding factor 2,
SCF	SKP/CUL1-F-box
SH2	Src 2 homology
SHP2	non-receptor PTP; encoded by the *Ptpn11* gene
STYX	phosphoserine/threonine/tyrosine-interacting protein
STYXL1	STYX-like-1
STYXL2	STYX-like-2
TGFβ1	transforming growth factor beta 1
XLCNM	X-linked centronuclear myopathy
ZEB	Zinc finger E-box-binding homeobox 1
